# Preparation, Characterization, and Unexpected Cathodoluminescence of Polycrystalline TlPbI_3_

**DOI:** 10.1021/acs.inorgchem.5c06084

**Published:** 2026-04-02

**Authors:** Maurice Conrad, Jonas Grill, Stephan Böhringer, Thomas Schleid, Michael Saliba

**Affiliations:** † Institute for Photovoltaics, 9149 University of Stuttgart, Pfaffenwaldring 47, 70569 Stuttgart, Germany; ‡ Helmholtz Young Investigator Group FRONTRUNNER, IMD-3 Photovoltaics, Forschungszentrum Jülich, 52425 Jülich, Germany; § Institute for Inorganic Chemistry, University of Stuttgart, Pfaffenwaldring 55, 70569 Stuttgart, Germany

## Abstract

A polycrystalline powder of orange-red TlPbI_3_ was prepared from equimolar amounts of TlI and PbI_2_ by solid-state synthesis in an evacuated silica ampule at 400 °C. This reaction was analyzed for the first time by differential scanning calorimetry: During the first temperature cycle, it was revealed that small amounts of precursors sublimate before the reaction mixture melts congruently and recrystallizes, forming the title compound. During the second temperature cycle, the melting point for the title compound was determined to be 345 °C. Rietveld refinement of room-temperature powder X-ray diffraction data shows that orthorhombic TlPbI_3_ crystallizes in the space group *Cmcm* with the lattice parameters *a* = 462.16(4), *b* = 1487.12(15), and *c* = 1181.43(11) pm for *Z* = 4. Optical studies by UV-Vis diffuse reflectance spectroscopy result in an absorption edge at λ_abs_ = 568 nm and an experimentally determined band gap at *E*_g_ = 2.23 eV for polycrystalline TlPbI_3_. Temperature-dependent cathodoluminescence studies on the polycrystalline sample show a broad signal at lower temperatures, which is blue-shifted by roughly 180 nm compared with previous reports.

## Graphical abstract

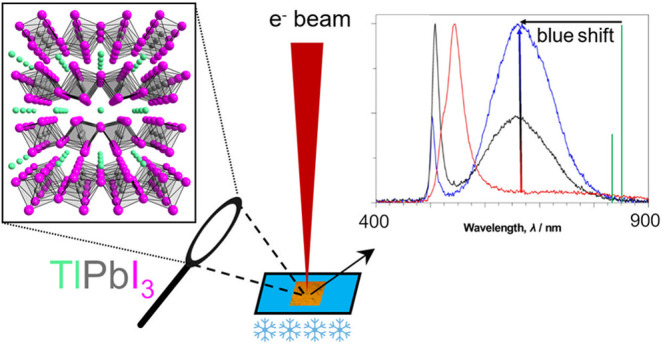

## Introduction

Scintillators convert high-energy into low-energy photons and thus are used in radiation detectors and medical imaging devices, such as computer tomography (CT) or positron emission tomography (PET) scanners.^1,2^ The requirements for scintillating materials are demanding, including among others^3^ an elevated attenuation power for high-energy photons. Depending on the energy of the incident photons, either the photoelectric effect (low energies, *E* < 100 keV),^4,5^ Compton scattering (mid energies, *E* ≈ 0.1–10 MeV),^6,7^ or electron-positron pair production (high energies, *E* > 10 MeV)^8^ are the prevalent absorption mechanisms. Since the absorption power is improved with increasing density *ϱ* and effective nuclear charge *Z*, materials exhibiting high values for ϱ and *Z* are particularly suited for scintillation.^9,10^ This encompasses, e.g., CsI:Tl (thallium-doped cesium iodide)^11^ or LYSO:Ce (cerium-doped lutetium yttrium oxide ortho-oxosilicate, Lu_1.8_Y_0.2_SiO_5_:Ce (= B-type Ln_2_O­[SiO_4_])^12,13^ ).

Especially, TlPbI_3_ can be identified as an effective inorganic scintillator with both a high density of ϱ = 6.49 g·cm^–3^ and a high average effective nuclear charge of *Z*_avg_ = 64.4.^14^ Its *Z*_avg_ value even reaches the theoretically achievable limit, since thallium (*Z* = 81) and lead (*Z* = 82) are the heaviest stable (nonradioactive) elements within the periodic table. Also, iodine (*Z* = 53) is the highest *Z* element to still form mononuclear anions when disregarding the very sensitive and uncommon aurides (Au^–^, *Z* = 79).^15,16^

Based on previous structural reports,^17,18^ TlPbI_3_ is considered a perovskitoid, i.e., its orthorhombic crystal structure is closely related to the cubic perovskite-type structure, mostly differing in the distorted, tilted, and partly edge-connected [PbI_6_]^4–^ octahedra due to the small radius of the Tl^+^ cation. Single-crystalline TlPbI_3_ has previously been investigated as a (room temperature) radiation detector.^14,19,20^ This includes works on physical and chemical properties, electronic structure featuring a wide band gap of around 2.25 eV,^18,21^ single-crystal growth,^18^ and quality,^20^ as well as thermal^14^ and optical properties, such as cryo-photoluminescence.^14,19^

However, to the best of our knowledge, there are no comprehensive reports of polycrystalline TlPbI_3_ yet. This may be due to the challenging synthesis, concerns for toxicity, and because the main focus of previous studies was primarily placed on single-crystalline TlPbI_3_. Here, we report the preparation and characterization of polycrystalline TlPbI_3_ including a calorimetric reaction to trace its formation. We also compare our obtained data with previous reports on single-crystalline TlPbI_3_ while focusing on its optical properties.

## Materials and Methods

**Caution!** Thallium-containing compounds are highly toxic and should therefore be handled with great care.

### Materials

During the investigations, the following materials were used as bought without further purification:

TlI, p.a., Merck, and PbI_2_, 99.99 % trace metals basis, TCI.

### Synthesis

In an argon-filled glovebox (GS Glovebox Systemtechnik, Germany, H_2_O and O_2_ < 0.2 ppm), equimolar amounts of thallium­(I) iodide TlI (126 mg, 0.38 mmol) and lead­(II) iodide PbI_2_ (176 mg, 0.38 mmol) were homogenized and transferred into a silica glass ampule, which was flame-sealed under vacuum. Afterward, the sealed silica ampule was transferred to a muffle furnace. The reaction mixture was heated from RT to 400 °C with 5 K·min^–1^, held at 400 °C for 6 h and subsequently cooled back to RT with a rate of 0.5 K·min^–1^. The occurring solid-state reaction illustrated in Scheme 1 (*bottom*) yielded phase-pure red powder of TlPbI_3_ (Scheme 1, *top*).

**Scheme 1:**
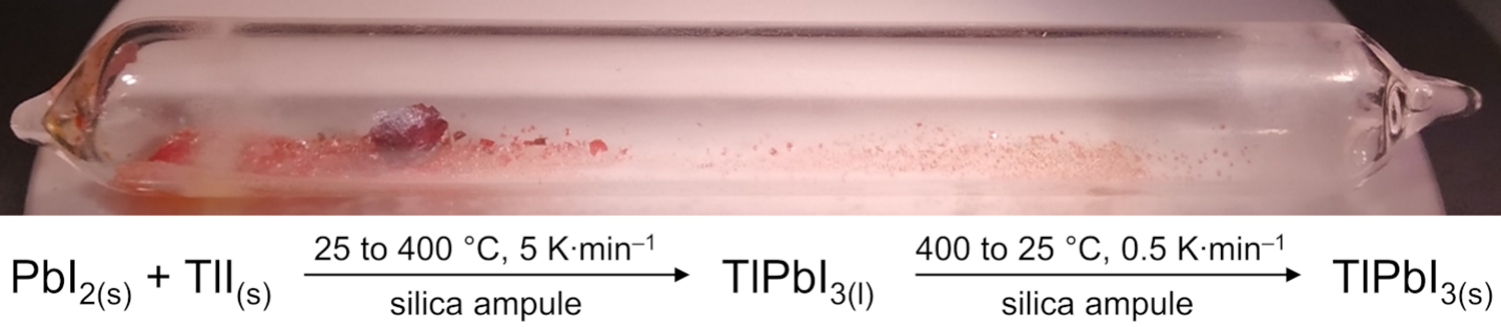
Solid-State Reaction (*Bottom*) Forming Red TlPbI_3_ in a Flame-Sealed Evacuated Silica Ampule (*Top*)

### Phase Analysis

Room-temperature powder X-ray diffraction studies were performed on a lab-scale powder X-ray diffractometer in *Debye-Scherrer* geometry (Stoe & Cie, Darmstadt, Germany) equipped with a Cu-*K*_α_ radiation source (λ = 154.06 pm), a curved *Johansson-*type germanium(111) monochromator, and a linear PSD detector. The sample was introduced into an argon-filled glovebox (GS Glovebox Systemtechnik, Germany, O_2_ and H_2_O ≤ 0.2 ppm) and homogenized by grinding in a mortar. Afterward, small amounts of the now reddish-orange TlPbI_3_ powder were sealed between two pieces of polyethylene terephthalate (PET) foil, which were coated with vacuum grease to ensure an airtight seal. Subsequently, they were inserted into a transmission holder, which was mounted on a sample holder of the diffractometer and rotated throughout the experiment. The diffraction data were collected in a 2θ range from 10 to 90° with a step width of 0.01°.

Rietveld analysis was performed using TOPAS 7.0.^22^ The background refinement was performed with a 1/*x* function and a second-order Chebychev polynomial, and microstructural parameters (crystallite size and strain broadening) were refined to adjust peak shapes (Figure 1a). The crystallographic data and refinement parameters are given in Table S1.

**Figure 1:**
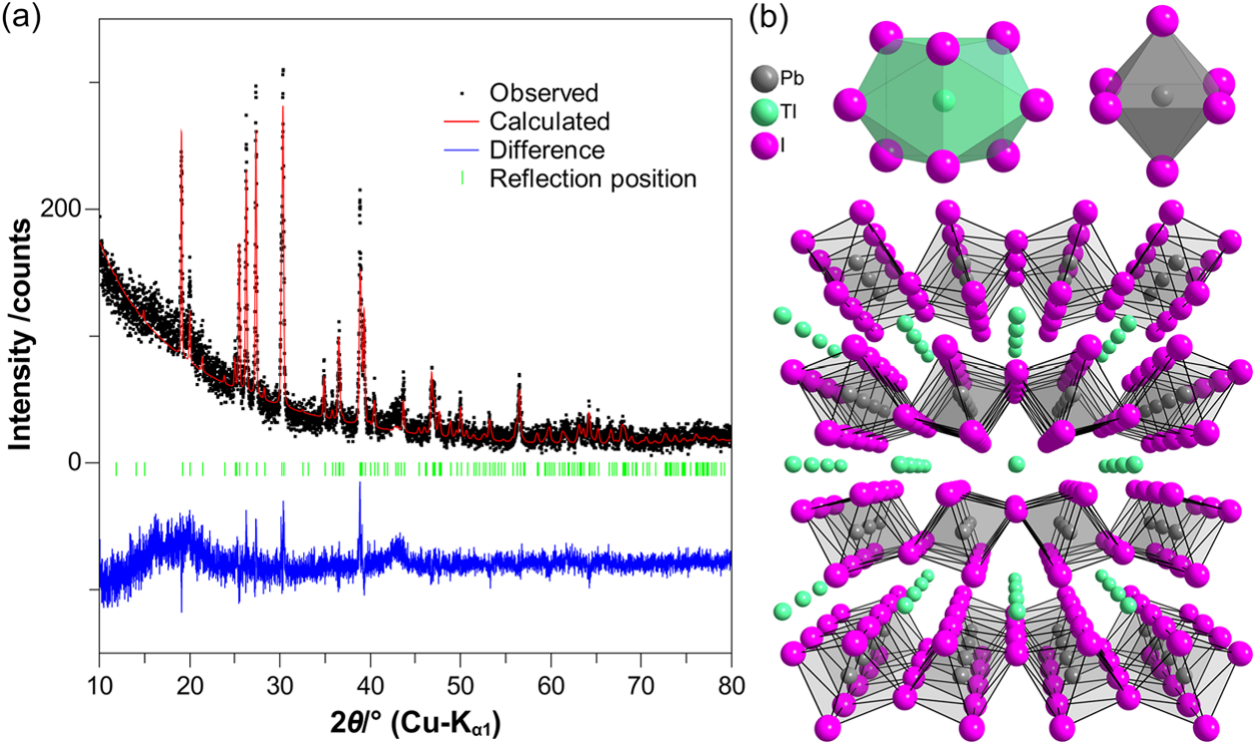
(a) Rietveld plot of polycrystalline TlPbI_3_ prepared by solid-state methods and (b) view at the orthorhombic crystal structure of TlPbI_3_ in central perspective (*bottom*), as well as the cationic coordination environments of Tl^+^ and Pb^2+^ (*top*).

### Elemental Analysis

To confirm the chemical composition of the sample, energy-dispersive X-ray spectroscopy (EDS) was performed on an SX-100 electron microprobe (Cameca, Paris, France) with an “Ultra Dry EDS Detector” (Thermo Fisher Scientific, Dreieich, Germany). A few crystallites of the sample were transferred to a conductive carbon pad and covered with a thin layer of evaporated carbon to ensure the conductivity of the sample. The EDS spectra of the sample (Figure S1) were collected with stationary measurements at different spots on the sample using an acceleration voltage of 15 kV, a beam current of 20 nA, and an irradiation time of 60 s.

### Reaction Tracing

To trace the solid-state reaction by differential scanning calorimetry (DSC), a NETZSCH DSC 404 (Netzsch Gerätebau, Selb, Germany) was used. Thus, 82 mg of the reaction mixture was transferred into a silica ampule, which was subsequently flame-sealed under vacuum. An empty sealed, evacuated silica ampule was used as reference. The sample was heated from RT to 500 °C with a rate of 10 K·min^–1^ and subsequently cooled back to RT with 10 K·min^–1^ in a constant argon gas flow. Two temperature cycles were recorded, the first one to trace the formation of TlPbI_3_ and the second one to investigate its thermal properties (Figure 2).

**Figure 2:**
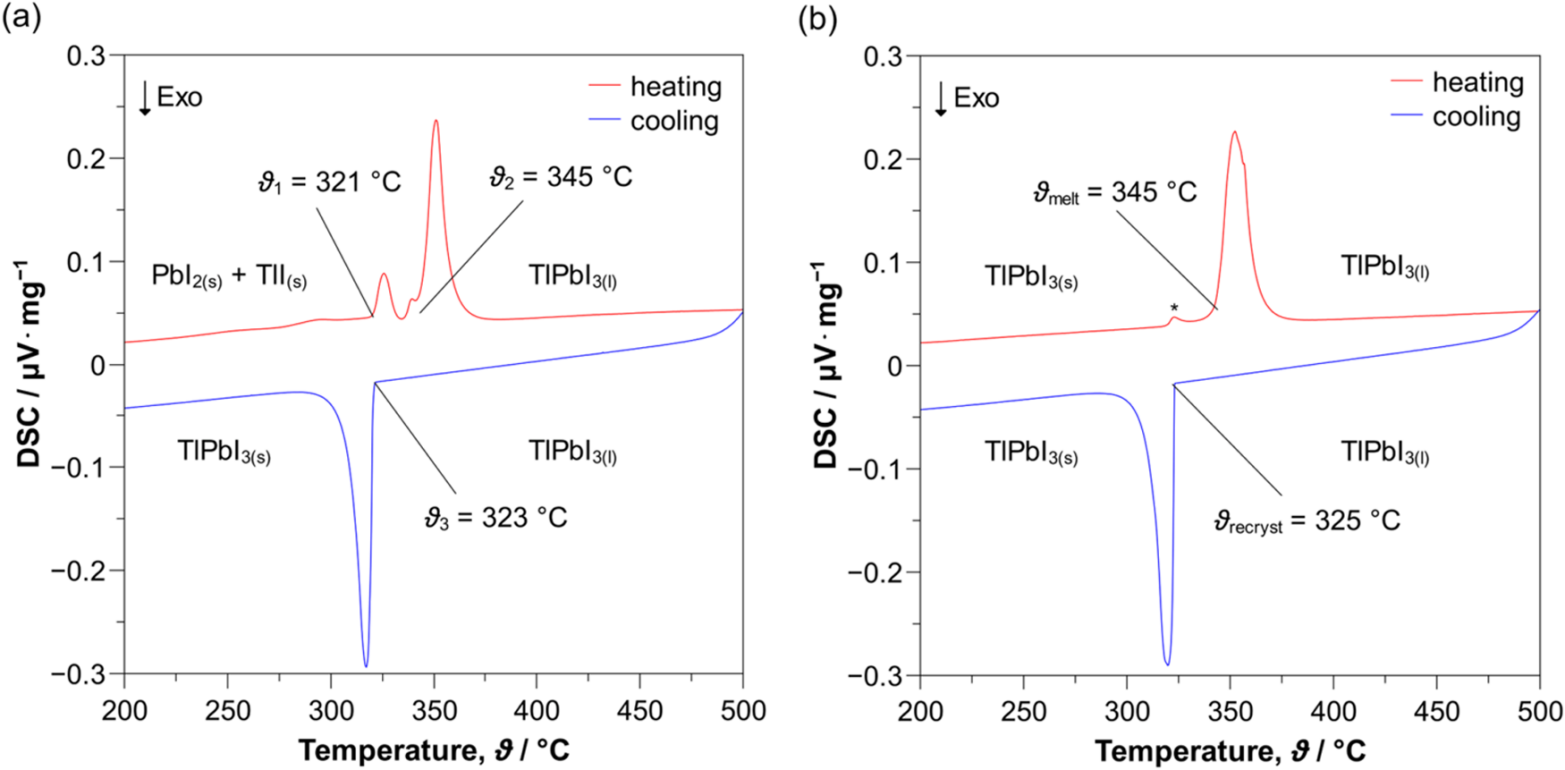
Thermal analysis of TlPbI_3_ in an evacuated silica ampule via DSC from 200 to 500 °C with a rate of 10 K·min^–1^. (a) Tracing its formation from the binary precursors PbI_2_ and TlI. (b) Congruent melting and recrystallization of TlPbI_3_. (*) Signal caused by traces of sublimated precursor materials.

### Optical Studies

#### Diffuse Reflectance Spectroscopy (DRS)

The diffuse reflectance sample holder cell of a TIDAS UV-Vis-NIR spectrophotometer (J&M Analytik AG, Essingen, Germany) was filled with about 5 mg of TlPbI_3_. Afterward, the diffuse reflectance spectrum (Figure S2) was recorded from 200 to 2000 nm (0.62–6.2 eV) with a spectral resolution of 4 nm against Ba­[SO_4_] as standard white reference. To determine the absorption edge, the reflection data was converted to a Kubelka-Munk absorption spectrum (Figure S3) using Kubelka-Munk theory.^23^ Afterward, the band gap was determined with Tauc plots (Figures 3 and S4).

**Figure 3:**
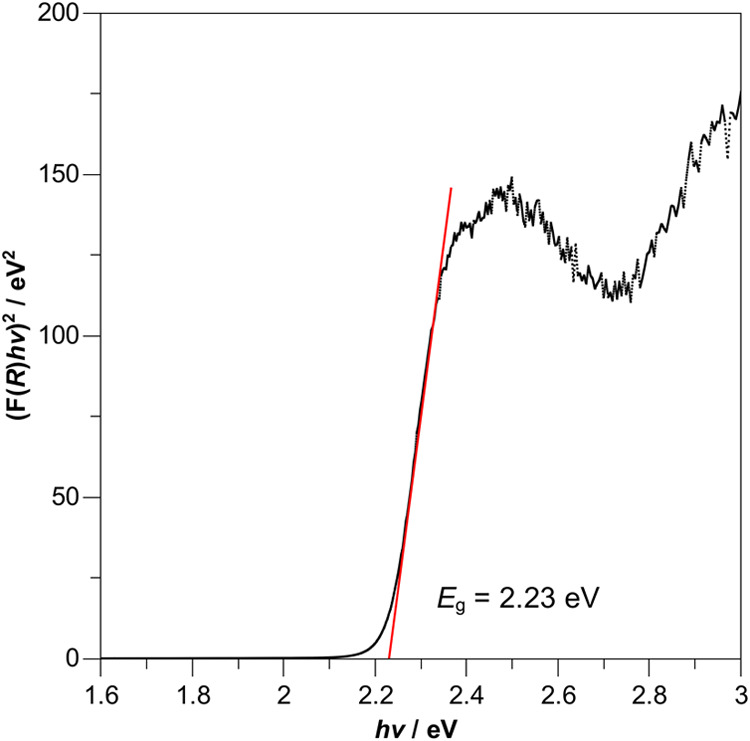
Tauc plot to determine the direct band gap *E*_g_ of polycrystalline TlPbI_3_ at room temperature.

#### Temperature-Dependent Cathodoluminescence

The cathodoluminescence (CL) measurements were conducted in a JSM-6460 scanning electron microscope (JEOL (Germany) GmbH, Freising, Germany) with a homemade detector based on a Hamamatsu S11156-2048-02 back thinned Si-CCD sensor cooled by a TEC to 263 K. A few mg of TlPbI_3_ were transferred to a conductive pad and subsequently mounted on the SEM sample holder. The vacuum within the chamber was kept at 4 × 10^–6^ mbar for the entire duration of the experiments. Afterward, the sample was cooled from RT to 170 K with a home-built liquid-nitrogen-based cryostat. The cryogenic stage consisted of a liquid-nitrogen dewar attached to the specimen chamber and a copper sample holder. Thermal connection to the sample holder was done via copper braids, a flexible connection to allow stage movement, and vibration isolation from boiling liquid nitrogen. To precisely control the temperature of the sample, an additional heater resistor was attached to the sample holder, which allows for variable temperatures. A photograph of the setup is shown in Figure S5 for clarity. The CL spectra of polycrystalline TlPbI_3_ were recorded from 300 to 900 nm at 170, 220, and 280 K by exciting the sample with 30 keV at 400 pA for 15 s. The excitation was done while scanning with 10 FPS over an area of approximately 50 × 50 mm^2^ to ensure a uniform measurement.

#### Thermochromic Behavior

A small amount of the title compound was sealed in an evacuated silica ampule and subsequently cooled to 195 K in a dry ice/acetone cooling bath and to 77 K in liquid nitrogen. Photographs at room light were taken at room temperature (RT), 195, and 77 K to capture the respective hues of the sample (Figure 4b).

## Results and Discussion

Powder X-ray diffraction reveals that the prepared sample of TlPbI_3_ is polycrystalline, and all reflections could be indexed with a single phase of TlPbI_3_. The perovskitoid adopts an orthorhombic crystal structure (Figure 1b, *bottom*) with the space group *Cmcm* and lattice parameters *a* = 462.16(4), *b* = 1487.12(15), and *c* = 11.8143(11) pm for *Z* = 4, known as KTmI_3_-type^24^ according to the ISCD, and matching well with previous structural reports.^17,18^ Layers of vertex- and edge-sharing [PbI_6_]^4–^ octahedra are held together by Tl^+^ cations in 8-fold bicapped trigonal prismatic I^–^ coordination. The unlike interatomic distances (*d*(Pb^2+^–I^–^) = 318 (2×) and 322 pm (4×), as well as *d*(Tl^+^–I^–^) 347 (2×), 361 (4×) and 397 pm (2×), Figure 1b, *top*) attest for a rather occult stereochemical lone-pair activity of the 6s^2^ cations Tl^+^ and Pb^2+^.

**Figure 4:**
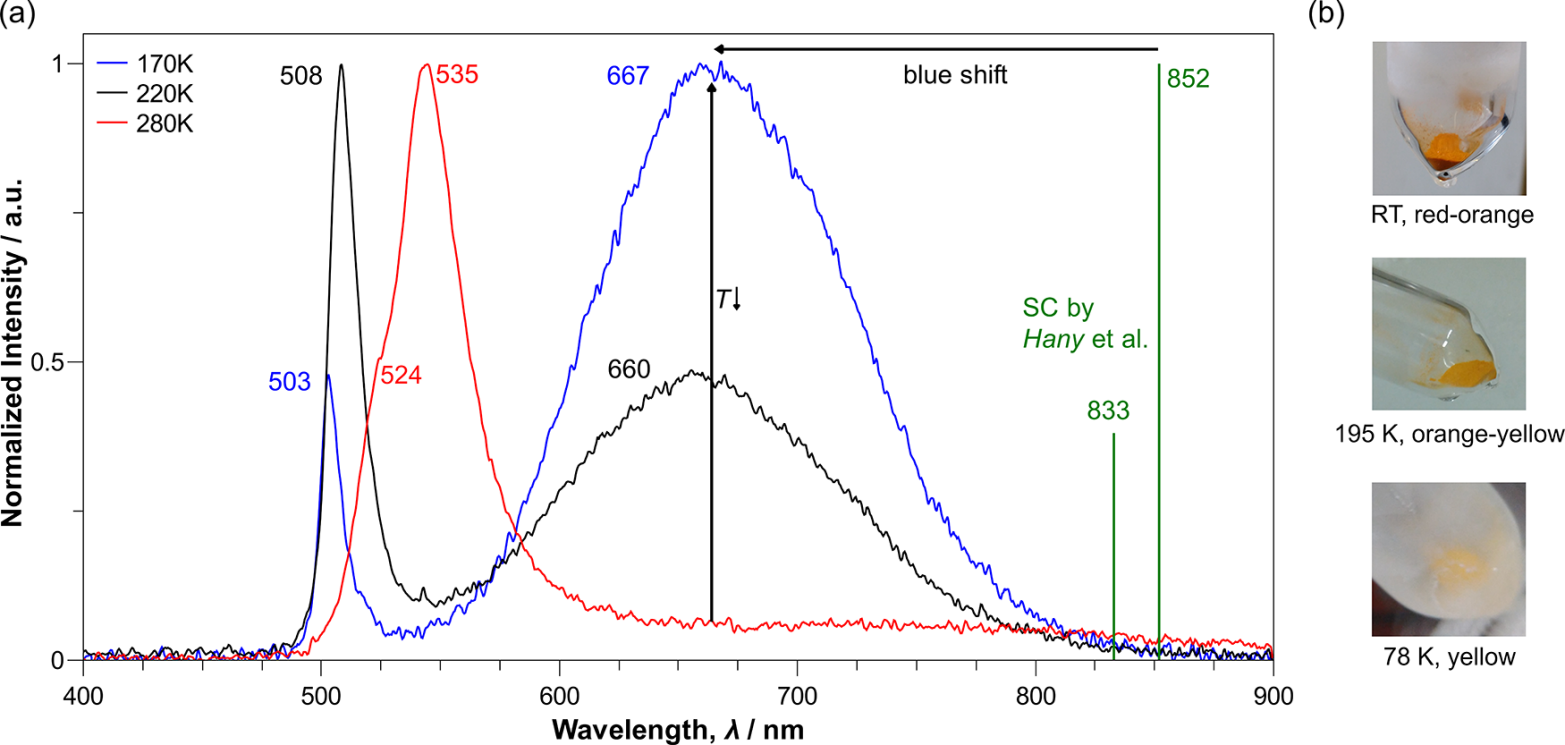
Low-temperature optical studies of polycrystalline TlPbI_3_. (a) Observed cathodoluminescence spectra after excitation with 30 kV at 170 K (blue), 220 K (black), and 280 K (red) and green lines indicating the maxima of the peaks in a previous study on single-crystalline (SC) TlPbI_3_.^19^ (b) Photographs at RT (*top*), 195 K (*mid*), and 78 K (*bottom*), illustrating the gradual color change from red-orange to yellow.

The fitted model reveals minor intensity mismatches compared to the recorded pattern (Figure 1a), which are probably caused by the preferred crystal orientation. However, due to the poor signal-to-noise ratio resulting from the high X-ray absorption of the material, the obtained data set does not allow reasonably for a refinement of the orientation. Therefore, all reflections exhibiting intensity mismatches were compared with patterns of the binary precursor materials PbI_2_ and TlI, as well as likely lead-poor byproducts, i.e., thallium lead iodides with the compositions Tl_3_PbI_5_,^25^ Tl_4_PbI_6_ ^17^, or Tl_6_PbI_10_ ^26^ revealing no matches and indicating the absence of crystalline byproducts.

Furthermore, EDS spectroscopy (Figure S1) confirms that apart from minor impurities of chlorine (approximately 1–2 %, Table S2), only thallium, lead, and iodine are present within the sample, therefore additionally supporting the assumption that no amorphous impurities are present.

To follow the formation of the ternary perovskitoid from its binary precursors, DSC measurements imitating reaction conditions (Figure 2a) were performed. During heating, two endothermal signals are observed at onset temperatures of ϑ_1_ = 321 °C and ϑ_2_ = 345 °C. The first small signal (ϑ_1_) is consistent with the well-known sublimation of PbI_2_ ^27^ with the possible addition of traces of TlI.^28^ This is supported by our observation of small amounts of yellow solid at the top of the silica ampule after the experiment. The second signal (ϑ_2_) exhibits a comparatively larger peak area caused by the congruent melting of the eutectic reaction mixture. Further heating to 500 °C does not show more thermal events. Upon cooling back to RT, only the recrystallization process of the melt (ϑ_3_ = 323 °C) and thus also the formation of the title compound is observed. During the second temperature cycle (Figure 2b), TlPbI_3_ melts congruently at ϑ_melt_ = 345 °C and recrystallizes at ϑ_recryst_ = 312 °C. Literature data for TlPbI_3_ show peak temperatures of about 360 °C for the melting and about 321 °C for the recrystallization process.^14^ This matches well with our peak temperatures at about 353 and 320 °C, respectively. The small differences are typical and due to different heating rates and the minutiae in referencing the silica ampules.

The diffuse reflectance spectrum of polycrystalline TlPbI_3_ was recorded at room temperature (Figure S2) and converted to a Kubelka-Munk absorption spectrum (Figure S3). It shows the absorption edge of polycrystalline TlPbI_3_ at around λ_abs_ = 568 nm in the range of visible light spectrum, whereas the Tauc plot (Figure 3) is used to estimate the direct band gap^14^ of the title compound to be *E*_g_ = 2.23 eV. A direct band gap was assumed since recent studies by Lin et al.^14^ and Yan et al.^21^ demonstrated the direct nature of the band gap with their electronic band structure calculations. However, since the band gap of single-crystalline TlPbI_3_ was previously also described as indirect,^18^ we also provide the Tauc plot of polycrystalline TlPbI_3_ for this case in Figure S4. The absorption edge and direct band gap determined in this work both match well with previously reported results for single-crystalline TlPbI_3_ (λ_abs_ = 570 nm,^19^ *E*_g_ = 2.25 eV^14^).

Temperature-dependent cathodoluminescence studies were performed on polycrystalline TlPbI_3_. The cathodoluminescence spectrum at 280 K (Figure 4a) reveals not only one peak close to the band gap at 535 nm (= 2.32 eV), which is typical for the closely related cubic halide perovskites,^29^ but also features an, at least for cubic perovskites, atypical distinct shoulder at 524 nm may be caused by structural changes. Upon cooling, this signal shifts toward higher energies possibly alongside an increase in the band gap, since the material is shown to be thermochromic, changing its color gradually from red-orange to yellow (Figure 4b), matching similar observations in the literature.^14^ At the same time, a second broad signal emerges at lower energies of around 665 nm. It is unexpected that the maximum peaks in the visible part of the spectrum, since previous cathodoluminescence studies on single crystals by Hany et al. observed it in the IR region at 833 and 852 nm.^19^ This means a significant blue shift of about 180 nm, despite identical optical absorption properties and structure data. This could be caused by the differences of our sample compared to the literature; i.e., our polycrystalline sample has a different ratio from bulk material to surface and grain boundaries. Another contributing factor could be differences in the preparation, purity, or origin of the base materials, potentially leading to either iodine-rich or iodine-poor domains within the structure similar to observations in Tl_2_Ba_2_CuO_6+δ_ polycrystals.^30^

Future studies are required to determine the influence of the possible traces of chloride within the sample that we observed in the EDS spectrum and to shed light on these subtle differences, e.g., directly comparing single- and polycrystalline TlPbI_3_ or trying different types of precursors and purification processes.

## Conclusions

A polycrystalline powder of orthorhombic TlPbI_3_ was prepared from binary halides (TlI and PbI_2_) by solid-state methods. Its formation was successfully monitored for the first time by DSC, revealing the sublimation of small amounts of halide precursor at around 321 °C before the congruent melting of the reaction mixture at 345 °C. The obtained crystallographic data of TlPbI_3_, its thermal properties, and its UV-Vis absorption data are consistent with previous reports. However, during low-temperature cathodoluminescence studies, a broad signal emerging at low temperatures is blue-shifted by about 180 nm compared to previous studies on single-crystalline samples. To understand the reason behind this contrast, further in-depth studies are required comparing both poly- and single-crystalline TlPbI_3_ that has been prepared in the same way.

## Supplementary Material



## Notes

EDS spectrum, results of the Rietveld refinement, original DRS data, Kubelka-Munk absorption spectrum, and Tauc plot for the indirect band gap case of polycrystalline TlPbI_3_ including a photograph of the cryo-CL setup (DOCX)

## ∥

Department of Energy and Petroleum Engineering, University of Stavanger, Stavanger 4021, Norway

## Abbreviations

CT: computer tomography
PET: positron emission tomography
DSC: differential scanning calorimetry
EDS: energy-dispersive X-ray spectroscopy
PET: polyethylene terephthalate
DRS: diffuse reflectance spectroscopy
CL: cathodoluminescence
TEC: thermoelectric cooler
ICSD: inorganic crystal structure database
RT: room temperature
SC: single-crystalline
