# Supplementary material for: Preparation, Characterization, and Unexpected Cathodoluminescence of Polycrystalline TlPbI3
Source: Inorg Chem. 2026 Apr 2;65(15):8431–6. doi: 10.1021/acs.inorgchem.5c06084 (PMC13100944; doi:10.1021/acs.inorgchem.5c06084)
Supplement: Supplementary file 1 [file ic5c06084_si_001.docx]

**Supplementary Information**

Preparation, Characterization and Unexpected Cathodoluminescence of Polycrystalline TlPbI_3_

Maurice Conrad^a^, Jonas Grill^a †^, Stephan Böhringer^a^, Thomas Schleid^c^ and Michael Saliba^a,b^*

1. Institute for Photovoltaics, University of Stuttgart, Pfaffenwaldring 47, 70569 Stuttgart, Germany.
2. Helmholtz Young Investigator Group FRONTRUNNER, IMD-3 Photovoltaics, Forschungszentrum Jülich, 52425 Jülich, Germany.
3. Institute for Inorganic Chemistry, University of Stuttgart, Pfaffenwaldring 55, 70569 Stuttgart, Germany.

* E-mail: michael.saliba@ipv.uni-stuttgart.de

**Table S1.** Rietveld refinement results of as-synthesized TlPbI_3_ at 293 K.

| Space group: *Cmcm*, *a* = 462.07(4) pm, *b* = 1486.98(15) pm, *c* = 1181.27(11) pm, *R*_wp_ = 17.30 %, *GoF* = 1.06 | | | | | | |
| --- | --- | --- | --- | --- | --- | --- |
| Atom | Wyckoff site | *x/a* | *y/b* | *z/c* | Occupancy | *B*_eq_ |
| Tl | 4*c* | 0 | 0.7482(5) | ^1^/_4_ | 1 | 1 |
| Pb | 4*a* | 0 | 0 | 0 | 1 | 1 |
| I1 | 8*f* | 0 | 0.3579(5) | 0.0599(6) | 1 | 1 |
| I2 | 4*c* | 0 | 0.0765(8) | ^1^/_4_ | 1 | 1 |


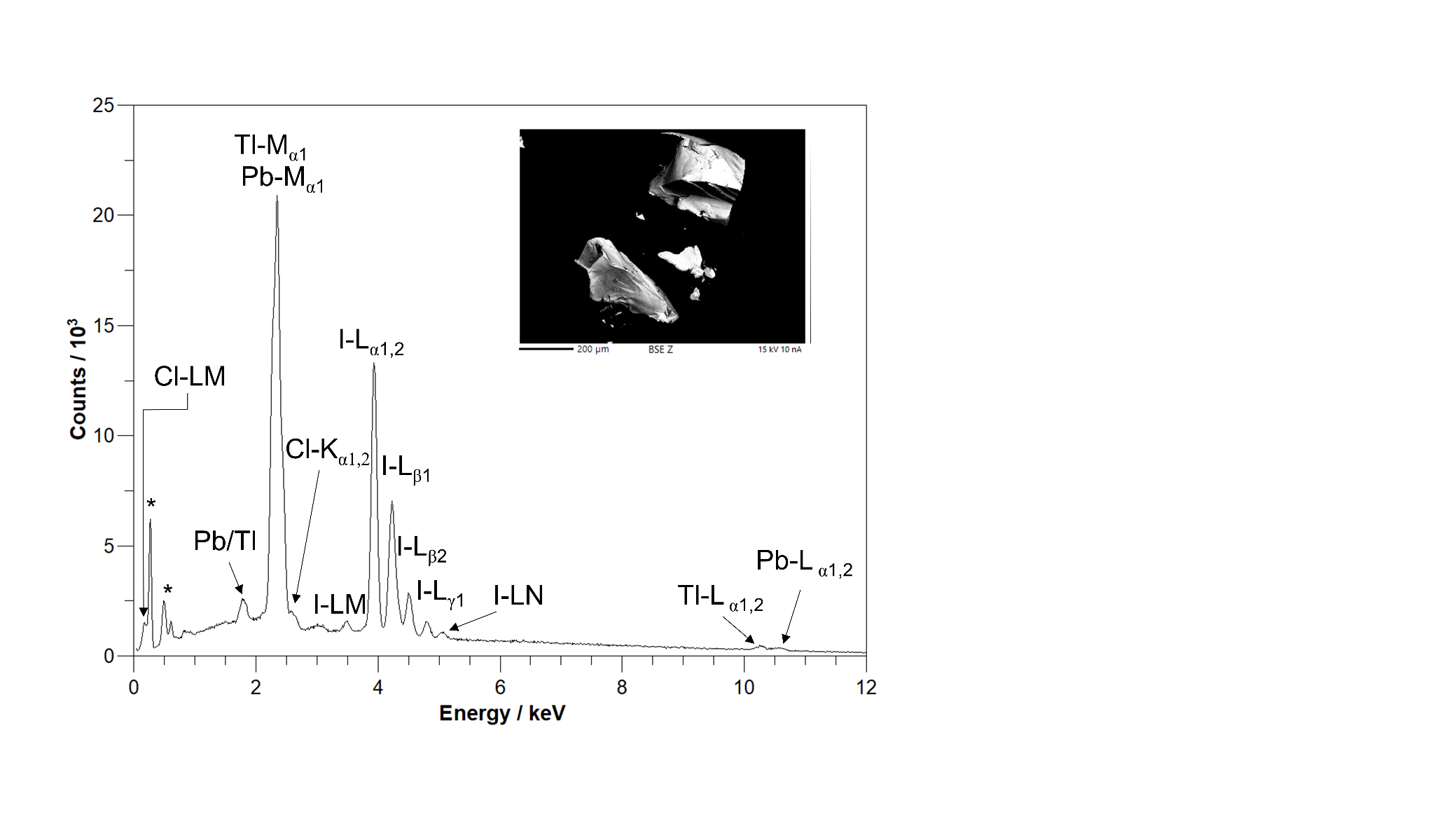


**Figure S1.** EDS spectrum of polycrystalline TlPbI_3_ at room temperature. * Lines due to sample preparation.

**Table S2.** Elemental distribution as calculated by Thermo Fisher NSS based on the EDS spectrum.

| **Element** | **Atom conc. / %** | **Error / %** |
| --- | --- | --- |
| Tl* | 20.5 | ±0.4 |
| Pb* | 16.7 | ±0.6 |
| I | 61.2 | ±0.9 |
| Cl | 1.6 | ±0.6 |

* Due to the large overlap of the M lines, the ratio of Tl to Pb cannot be determined more accurately.


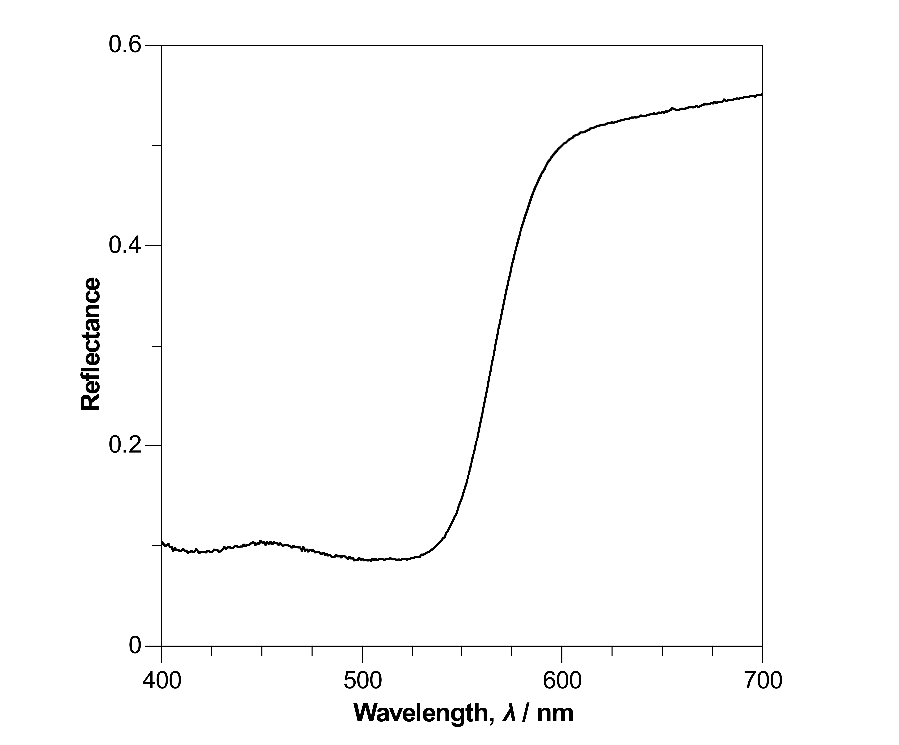


**Figure S2.** Diffuse reflectance spectrum of polycrystalline TlPbI_3_ at room temperature.


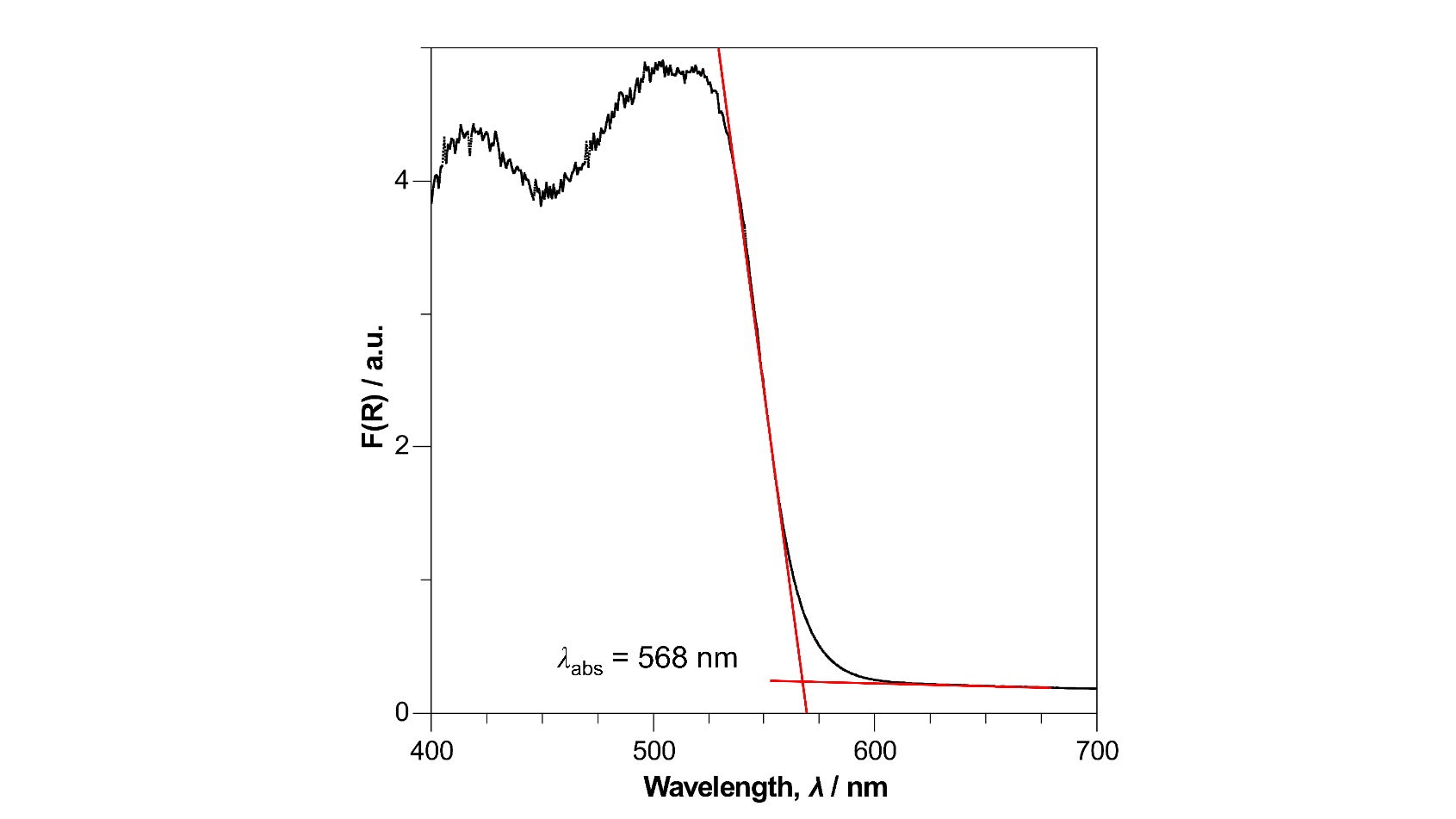


**Figure S3.** Kubelka-Munk absorption spectrum with absorption edge λ_abs_ of polycrystalline TlPbI_3_ at room temperature.


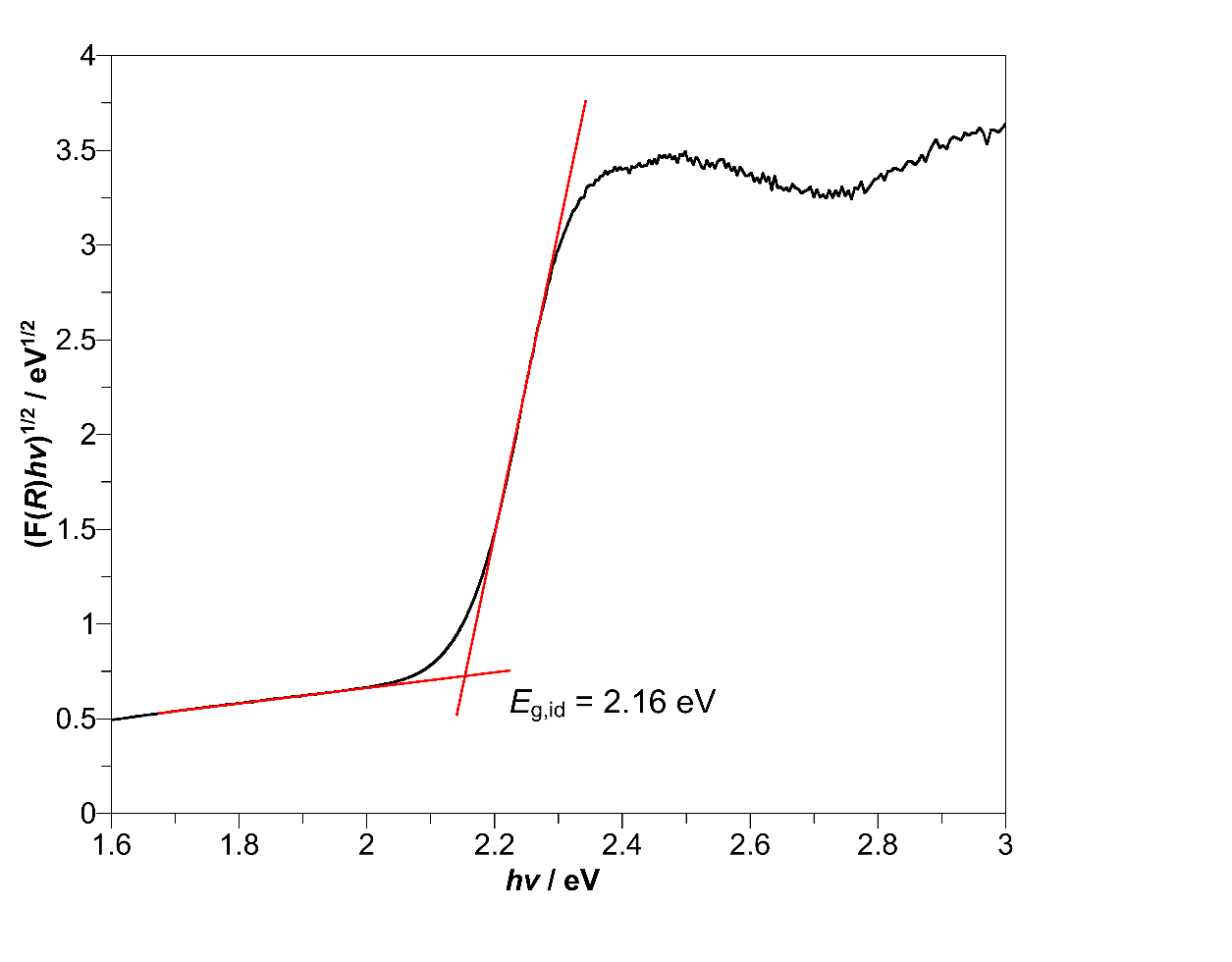


**Figure S4.** Tauc plot to determine the indirect band gap E_g,id_ of polycrystalline TlPbI_3_ at room temperature.


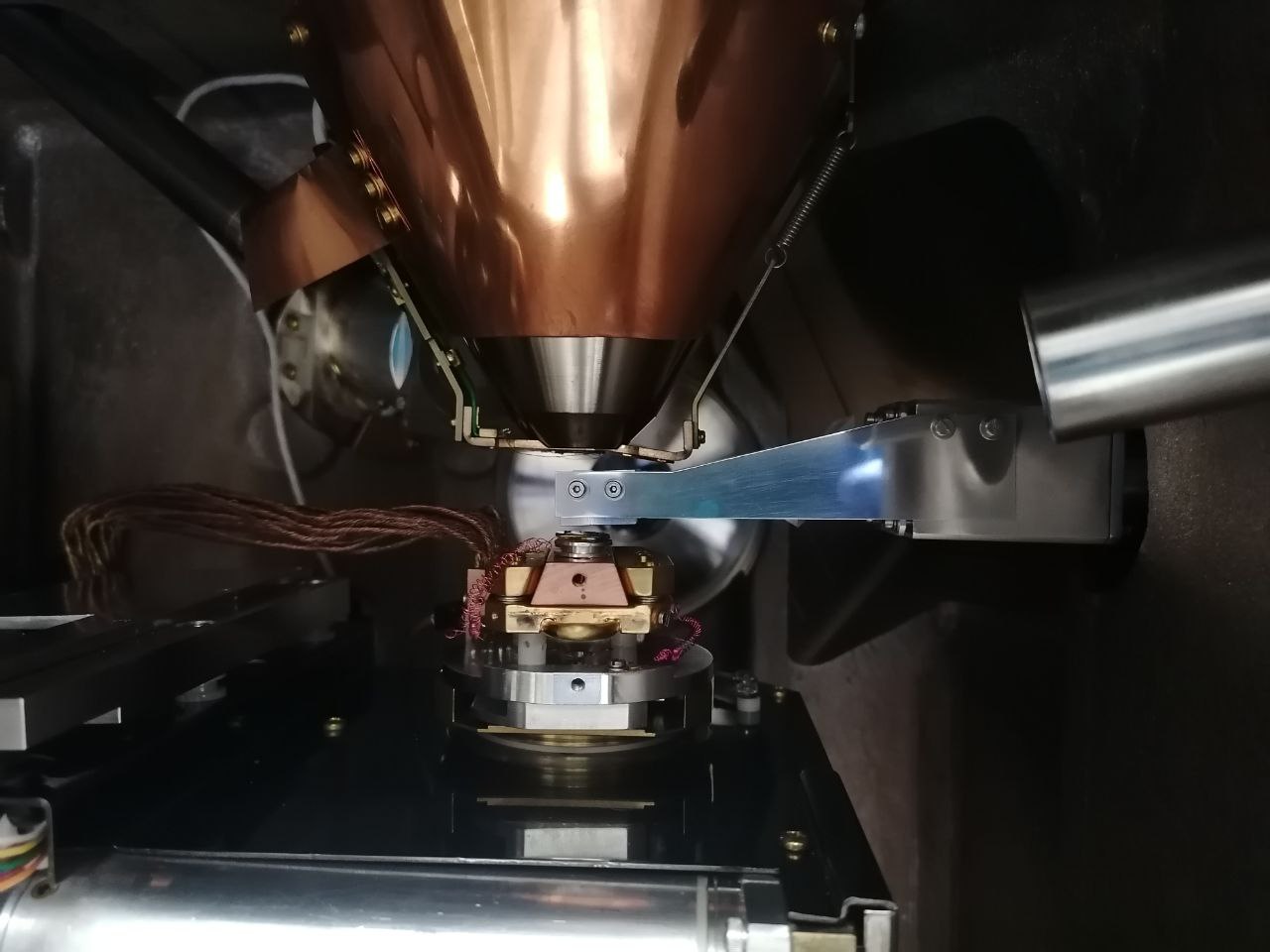


**Figure S5.** Photograph of the cryogenic stage with the CL collection mirror between sample and polepiece.
